# Starch-Based Hydrogel Nanoparticles Loaded with Polyphenolic Compounds of Moringa Oleifera Leaf Extract Have Hepatoprotective Activity in Bisphenol A-Induced Animal Models

**DOI:** 10.3390/polym14142846

**Published:** 2022-07-13

**Authors:** Hend Mohamed Hasanin Abou El-Naga, Samah A. El-Hashash, Ensaf Mokhtar Yasen, Stefano Leporatti, Nemany A. N. Hanafy

**Affiliations:** 1 Nutrition and Food Science Department, Faculty of Home Economics, Al-Azhar University, Nawag, Tanta P.O. Box 31732, Egypt; hendaboelnaga47@gmail.com (H.M.H.A.E.-N.); samahel-hashash@azhar.edu.eg (S.A.E.-H.); ensafyasin.2066@azhar.edu.eg (E.M.Y.); 2 Cnr Nanotec-Istituto di Nanotecnologia, Via Monteroni, 73100 Lecce, Italy; stefano.leporatti@nanotec.cnr.it; 3 Nanomedicine Group, Institute of Nanoscience and Nanotechnology, Kafrelsheikh University, Kafr El Sheikh 33516, Egypt

**Keywords:** starch, Bisphenol A, Moringa leaf extract, encapsulating Moringa leaf extract

## Abstract

Bisphenol A (BPA) is an xenoestrogenic chemical used extensively in the fabrication of baby bottles, reusable plastic water bottles and polycarbonate plastic containers. The current study aims to investigate the hepatoprotective activity of *Moringa oleifera* Lam leaf extract (MOLE) and hydrogel NPs made of starch-MOLE-Bovine Serum Albumin (BSA) against Bisphenol A-induced liver toxicity in male rats. Fabrication and characterization of hydrogel NPs formed of starch-MOLE-BSA were investigated using FTIR, TEM, zeta potential, UV-visible spectroscopy and fluorescence spectrophotometer. The potential efficacy of hydrogel NPs was studied. Compared to the results of control, the level of liver function, oxidative stress markers and lipid profile status were remodulated in the groups treated with MOLE and hydrogel NPs (Encap. MOLE). Meanwhile, the administration of MOLE and Encap MOLE significantly increased antioxidant activity and decreased the level of apoptotic pathways. Heme oxygenase (HO)-1 and growth arrest -DNA damage-inducible gene 45b (Gadd45b) were also regulated in the groups treated with MOLE and Encap. MOLE compared to the group which received BPA alone. In the present study, MOLE and hydrogel NPs led to remarkable alterations in histological changes during BPA administration. Overall, MOLE has a potential antioxidant activity which can be used in the treatment of liver disorders.

## 1. Introduction

Liver damage is one of the main problems associated with the exposure to harmful materials [1]. The main function of the liver is to filtrate blood from the digestive tract, before passing it to the rest of the body. Thus, the liver can detoxify chemicals and metabolize drugs [2]. For this reason, if the liver is exposed to chemical materials, this can cause physiological disorder [2]. Bisphenol A (BPA) is widely used as a coating layer inside canned foods and beverages, baby bottles, packaged baby formula, pre-packed foodstuffs and containers that were made for food storage in the home. Additionally, BPA can also be found in dental prosthetics and sales receipts that use thermal paper [3]. BPA can leak out of epoxy resin and further diffuse into canned foods, ultimately entering the human body [4]. Furthermore, BPA can interfere with estrogen levels (normal function) within the human body [5]. Therefore, BPA impacts the function of androgen receptors, thyroid hormone receptors and other endocrine system signaling pathways [6]. Previous literature has shown that a small amount of bisphenol can have a significant effect on biological systems because of its ability to change the pathological condition of hormonal signaling pathways [7,8]. This condition can increase psychological and metabolic disorders in children, reproductive disorders in adults and neoplasms resulting from a weakened immune system after their exposure to BPA [9].

Currently, a lot of research has focused on natural compounds extracted from medicinal plants which could assist in the treatment of pathological conditions [10]. *Moringa oleifera* Lam (MOL) contains many bioactive and antioxidants materials which may overcome oxidative stress and degenerating diseases [11,12]. *Moringa oleifera* Lam leaves have many biological activities that can be used in the prevention of cardiovascular diseases [13], such as immune boosting agent and hypotension [14], cholesterol lowering, diuretic and antiulcer properties [15] and antioxidant activity [16]. These properties are mostly useful for protecting the liver against hepatotoxin-induced toxicity [17]. However, the bioactive compounds are highly susceptible to degradation, and this may decrease the antioxidant activity present in MOL. To prevent these limitations, the utilization of the micro/nanoencapsulation technique is necessary [18]. MOL extracts are highly unstable and susceptible to oxidation; they also show limited solubility in water and low bioavailability [19].

The application of nanotechnology in the food industry has grown in the last few decades, causing the use of micro/nanoparticles which are consumer safe [20]. Therefore, the current study aimed to investigate the protective effect of *Moringa oleifera* Lam leaf extract (MOLE) and encapsulating *Moringa oleifera* Lam leaf extract (Encap. MOLE) against BPA toxicity in an experimental animal model.

Starch-based hydrogel NPs are considered a novel approach in the field of drug delivery systems due to the fact that the application of hydrogels in the pharmaceutical industry provides high drug stability, high loading capacity, large chemical interaction surface, drug protection and controlled drug release. Additionally, hydrogel increases mucoadhesive properties leading to improved penetration and cellular uptake.

Starch is a natural biopolymer which can be used as a gelling, thickening or stabilizing mechanism in various industrial and pharmaceutical applications. Amylase and amylopectin are the two main components of starch that form granule assembly. Hanafy succeeded in insulating starch from corn flour by using alkaline hydrolysis and used it to encapsulate anthocyanin inside its moieties. In the current study, an alkaline gelatinization method was used, where the alkaline-treated starch could form a network, generating hydrogels [21]. Then, Moringa oleifera Lam leaf extract was incorporated into the moieties of starch, which were coated with bovine serum albumin (BSA). The nanosized structure was assembled in hydrogel NPs [22].

BSA is a water-soluble molecule which can interact directly with any other organic or inorganic material by different types of chemical interactions. Owing to its charged amino acids, the electrostatic adsorption of negatively or positively charged molecules can be obtained. In this case, substantial amounts of drug can be incorporated within the particle, due to different albumin-binding sites [23].

In the current study, starch-MOLE-BSA was used to form micellar assembly and to investigate the hepatoprotective activities of *Moringa oleifera* Lam leaf extract. For this reason, the parameters were used as follows: The levels of liver function for serum alanine aminotransferase (ALT), aminotransferase (AST), alkaline phosphatase (ALP), total protein, albumin, lipid profiles (total cholesterol, triglyceride, phospholipid), antioxidant markers (liver glutathione (GSH), superoxide dismutase enzyme (SOD)), apoptotic markers (Caspase-3 and Bax), cell homeostasis genes (HO-1 and GADD45B) and histopathology (hematoxylin and eosin (H+E) and Masson trichrome stain).

## 2. Materials and Methods

### 2.1. Plant Materials and Chemicals

*Moringa* (*Moringa oleifera* Lam) leaves were obtained from the Ministry of Agriculture, Kafr El Sheikh Governorate, Egypt. BSA and Bisphenol A (CAS 80-05-7 Sigma–Aldrich Co., St. Louis, MO, USA) were purchased from Sigma Company, Egypt. Trizol Reagent (Invitrogen, Carlsbad, CA, USA) and (3,3′,5,5′-Tetramethylbenzidine) (TMB) were purchased from Sigma company. The kits of biochemical tests were purchased from Gamma Trade Company for biochemicals, Cairo, Egypt. All other required chemicals were obtained from Elgomhouria Company for trading Drugs, Chemicals and Medical Appliances, Cairo, Egypt.

### 2.2. Preparation of MOLE

Fresh MOLs were washed under running water, then they were shade dried at room temperature. After that, they were crushed into homogenized powder in the blender and stored at room temperature in a closed brown glass container in the dark until used. The extraction of *Moringa oleifera* Lam Leaves was prepared by mixing 10 g of crushed *Moringa oleifera* Lam Leaves in 45 mL of ethanol (70%), which was stirred for 1 h at 70 °C. Upon completing the extraction, 5 mL acetone was added and the stirring continued for an additional 30 min. The solution was further centrifuged and the supernatant was then collected [24].

### 2.3. Fabrication of Encapsulated MOLE

Starch (0.5 g) was suspended into 50 mL distilled water. Then, a few drops of NaOH (1N) was added dropwise, forming the gelatinized solution under mechanical stirring power. Then, it was centrifuged at 5000 rpm for 5 min and the starch supernatant was removed and kept at room temperature [25].

The extracted starch (20 mL) was completed to 80 mL with distilled water and then 1 mL MOLE was added. The mixture was then stirred for 20 min and 50 mg/50 mL of bovine serum albumin (BSA) solution was added. The stirring then continued for a further 30 min. After that, nanoparticles were dialyzed in the dialysis bags (Molecular weight cutoff of 12 to 14 kDa). Then, the sample was kept at −20 °C for lyophilization (Labconco, Freezone 1 L) at 5 mm Hg at −50 °C for 72 h. The lyophilized powder was stored at −20 °C until analysis [26].

### 2.4. Extraction and Identification of Phenolic Compound Using HPLC

Phenolic compounds were identified and measured by high-performance liquid chromatography (HPLC) according to a previously reported method [27].

### 2.5. Characterization Techniques of Nanoparticles

#### 2.5.1. Measurement of Zeta Potential

Dynamic light scattering of the prepared nanoparticle formulations as well as their charges were investigated using a Zeta-sizer (Brookhaven). In total, 1 mg of NPs was diluted into 10 mL distilled water at pH 7.4 for DLS and zeta potential measurements. For the analysis, an average of five successful runs were carried out at 25 °C.

#### 2.5.2. Transmission Electron Microscopy (TEM)

The Encap. MOLE nanoparticles (Encap. MOLE NPs) were acquired by using a TEM (JEOL 2100, Tokyo, Japan). The diluted samples were dropped upon a carbon-coated copper grid and the excess was drawn of. The samples were left to dry for 5 min and then images were acquired.

#### 2.5.3. Absorbance and Fluorescence Spectrophotometers

UV-vis spectroscopy was used to assess the wavelength and to identify the specific absorption for MOLE, BSA and Encap. For MOLE, 1 mL of the samples was added to 3 mL of distilled water, which was then measured using the UV-Vis spectrophotometer (Jasco V-770 UV Visible Absorbance Spectrophotometer) in the 200–800 nm wavelength range. Additionally, the fluorescence spectrophotometer was used for the same procedure. The obtained results were analyzed utilizing Origin 8.

#### 2.5.4. Fourier Transform Infrared Spectroscopy (FTIR)

FTIR experiments were carried out using JASCO Fourier Transform Infrared Spectrometer (Japan, model no. AUP1200343) to detect the surface molecular structures in the range of 400–4000 cm^−1^. Few dried samples of starch, BSA, MOLE and Encap. MOLE were grounded with KBr into homogenous powder and pressed into a suitable tablet with good thickness. For all the tests, at least three scans were recorded for different regions on the samples and the representative spectra were analyzed [28].

### 2.6. Animals and Ethical Approval

A total of 36 adult Sprague Dawley male albino rats weighing 200 ± 5 g were obtained from Helwan Farm, an animal colony, VI Org., Cairo, Egypt. They were kept in polypropylene cages at a room temperature of 22 ± 1 °C, relative humidity of 50 ± 20% and under a 12 h light/dark cycle. Water was supplied *ad libitum*. They were left to adjust to the laboratory conditions for one week before beginning the experiment. This study was approved by the Ethics Committee of Kafrelsheikh University [25].

### 2.7. Experimental Diet

Pelleted food was purchased to feed the rats from the Agricultural Development Company, 6-October City, Giza Governorate, Egypt. The food consisted of sunflower oil (15%), concentrate mixture 45% (10%), yellow corn (49%), soybean meal 44% (11%), wheat bran (10%), molasses (3%), common salt (0.5%), ground limestone (0.2%), dicalcium phosphate (0.1%), lysine (0.2%), dl-methionine (0.7%) and mineral-vitamin premix (0.3%).

### 2.8. Experimental Design and Sampling

#### 2.8.1. Concentration of BPA

The dose of BPA (50 mg/kg/daily) was chosen according to many previous studies [29,30].

#### 2.8.2. Experimental Design

Male rats were randomly assigned [31,32,33] to 6 groups of 6 rats for 4 weeks as follows: group I: received normal saline and kept as an untreated group (control group); group II: given BPA (50 mg/kg/day); group III: given MOLE (50 mg/kg/day); group IV: given MOLE (50 mg/kg/day) + BPA (50 mg/kg/day); group V: given Encap. MOLE (50 mg/kg/day); and group VI: given Encap. MOLE (50 mg/kg/day) + BPA (50 mg/kg/day). BPA, MOLE and Encap. MOLE were given orally. Rats in groups four and six (IV and VI) received MOLE and Encap. MOLE two hours after BPA administration.

Following this, animals were fasted overnight, then anesthetized by intraperitoneal injection of 70 mg/kg pentobarbital sodium. Blood samples were collected from the hepatic portal vein of the rats and placed into dry, clean centrifuge tubes. Sera were carefully separated by the centrifugation of blood samples (3000 rpm for 10 min) at room temperature, then placed into dry, clean Eppendorf tubes and kept frozen at −20 °C for biochemical determinations. Livers were carefully dissected, washed in ice-cold saline (0.9 g/100 mL), and dried using filter paper. After that, a specimen from each liver was immersed in buffered neutral formalin solution (10%) for histopathological examination, while other specimens were stored at −80 °C for other biochemical and molecular investigations [25,26,27].

### 2.9. Homogenization of Liver Tissue

To prepare the liver tissue homogenate, 1 g of liver tissue was removed and cut into small pieces, then it was homogenized using 4710 Ultrasonics Homogenizer (Cole-Parmer Instrument Co., Salisbury, NC, USA) in (1.15 g/100 mL) KCl (ice cold solution) in the presence of 50 mmol/L potassium phosphate-buffered solution (pH 7.4). The homogenized tissues were further centrifuged at 4000 rpm at 4 °C for 5 min. After that, supernatants were used in the experiments [34].

### 2.10. Biochemical Indices in Liver Tissue Homogenate and Sera

#### 2.10.1. Antioxidant Enzymes

In the liver tissue homogenate, the activities of reduced glutathione (GSH) and superoxide dismutase (SOD) were measured following referenced methods [35,36].

#### 2.10.2. Lipid Profile

Total cholesterol (TC) and triglycerides (TG) were determined in the homogenate of the liver tissue according to the methods described by Richmond [37] and Jacobs and VanDenmark [38], respectively. Additionally, the concentration of phospholipids (PhLs) was calculated in liver tissue homogenate according to the method of Ray et al. [39].

#### 2.10.3. Liver Function Biomarkers

In sera, the activities of liver enzymes, including aminotransferases (ALT and AST) and alkaline phosphatase (ALP), were determined following the methods of Reitman and Frankel [40] and Kind and King [41], respectively. In addition, albumin and total protein (TP) were determined by using the following referenced methods [42,43].

### 2.11. ELISA for Caspase-3 and Bax Detection

Enzyme-linked immunosorbent assay (ELISA) was used to measure the level of Caspase-3 and Bax [44]. Briefly, the homogenate of liver tissue and standards (100 μL) were added separately into the wells. Then, they were incubated for 2 h at 37 °C. The unreacted materials were washed and then 100 μL of biotin-conjugated antibody was added as a specific detector for Caspase-3 and Bax. After cleaning, 100 μL of avidin conjugated horseradish peroxidase (HRP) was added to the wells and then the samples and standards were incubated for 1 h at 37 °C, followed by the addition of 90 μL of TMB substrate solution. After that, incubation for 20 min was carried out at 37 °C to obtain proportional color to the amount of Caspase-3 and Bax. Then, the reaction was finished by adding stop reaction, then the microplate was tapped gently for thorough mixing and 450 nm was used. The activity of caspase-3 and Bax is expressed as ng/mL.

### 2.12. RNA Extraction

Total ribonucleic acid (RNA) was extracted by using a specific reagent called Trizol reagent following the standard’s protocol [45]. Complementary deoxyribonucleic acid (cDNA) was synthesized using a cDNA synthesis kit based on the standard’s protocol. The cDNA thermocycler was left at 37 °C for 30 min. qPCR procedure was run under three conditions, 95 °C for 5 min, 45 cycles at 95 °C for 30 s and 60 °C for 1 min. mRNA expression level was normalized into endogenous control (GAPDH). Then, the calculation was carried out as the relative differences between the control and treatment groups. Primers and probes for the qPCR were designed using Allele ID 6. All primers are listed in Table 1.

### 2.13. Histopathological Examination

Liver specimens were immersed in 10% phosphate-buffered neutral formalin (dehydrated, cleared in xylene), then the specimens were processed into paraffin blocks and cut off at 5 μm thickness. Sections were stained by normal histology routes using hematoxylin and eosin [46] or using Masson trichrome stain for collagen fibers [47]. Images were acquired using an inverted light microscope.

### 2.14. Biostatistics

The results were expressed as mean ± standard division of mean (SEM). Data were analyzed by SPSS version 20 using one-way analysis of variance (ANOVA), followed by Duncan’s test for comparison between different treatment groups. The data are shown as * *p* < 0.05 ** *p* < 0.01 and *** *p* < 0.001. The data are representative of at least three independent experiments.

## 3. Results

### 3.1. HPLC Identification and Quantification

**Figure 1:**
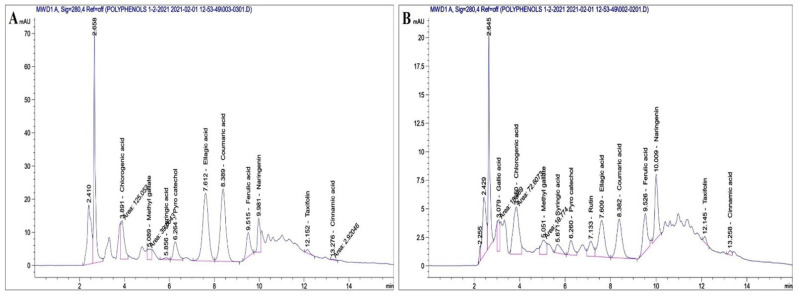
Quantification and identification of polyphenol and flavonoids content isolated from MOL (**A**) and were then encapsulated (**B**).

The composition of polyphenolic compounds extracted from MOLE and Encap. MOLE was determined by high-performance liquid chromatography (HPLC) and the results are listed in Table 2 and Figure 1.

It was found that the major polyphenolic compounds found in ethanolic extract of MOLE were ellagic acid (26 µg/mL). Chlorogenic acid was the second compound found (9.55 µg/mL), followed by coumaric acid, pyrocatechol and naringenin. Another seven compounds were found in small concentrations, ranging between 3.3 µg/mL (rutin) and 0.03 µg/mL (cinnamic acid) [48,49]. On the other hand, the major polyphenolic compounds in Encap. MOLE were found to be chlorogenic acid (5.54 µg/mL), followed by ellagic acid, naringenin, rutin, ferulic acid and gallic acid. Another six compounds were found in Encap. MOLE, but in very small concentrations, ranging between 1.08 µg/mL (coumaric acid) and 0.04 µg/mL (cinnamic acid).

### 3.2. Characterization

**Figure 2:**
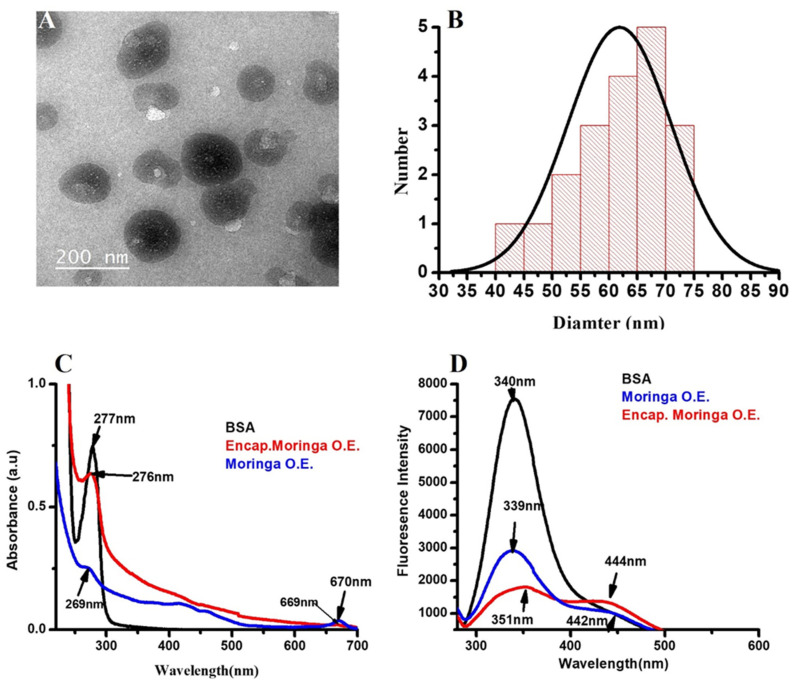
Characterization of MOLE NPs. (**A**) TEM image. (**B**) Quantification of NP diameter by using image J and Origin 8 program. (**C**) UV-Visible spectrophotometer for MOLE, BSA and Encap. MOLE. (**D**) Fluorescence spectrophotometer for fluorescence intensity of BSA, MOLE and Encap. MOLE.

**Figure 3:**
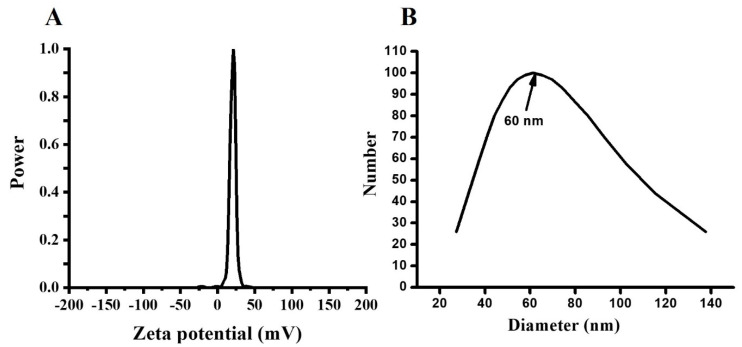
Zeta potential measurement of Encap. MOLE NPs (**A**). Nanosizer of Encap. MOLE NPs (**B**).

Starch is a natural biodegradable polymer which contains amylose and amylopectin as the main units of its components [50]. These two units are assembled in the shape of granules, the size ranging from 1 to 100 μm [51,52]. In the current study, TEM images showed spherical nanoparticles with diameters ranging between 40 and 75 nm. Their assembly confirms the successful formation of starch-MOLE-BSA NPs (Figure 2A). For instance, the two main components of starch may contribute to the formation of starch backbone, leading to the final structural shape of NPs. The UV visible spectrophotometer of the extract showed the absorption peak at 269 nm [53] and the characteristic absorbance peaks of MOLE were previously shown at 210 nm and 265 nm (π → π* transition of the aromatic conjugated ring), and at 330 nm (n → π* transition of hydroxyl groups (non-bonding electron) within the phenolic ring), while encapsulated MOLE peaked at 276 nm. This shift may be due to its interaction with starch components. Meanwhile, pure BSA had an absorption peak at 277 nm due to the weak absorption of tryptophan (Trp), aromatic amino acids phenylalanine (Phe), and tyrosine (Tyr), (Figure 2C). [54]. Additionally, MOLE and Encap. MOLE exhibited fluorescence intensity at (339–442 nm) and (351–444 nm), respectively. Zeta potential of Encap. MOLE showed potential surface charge at (21 mV) with good distribution (Figure 3). This result shows that they are capable of being stable drug carriers in humans.

**Figure 4:**
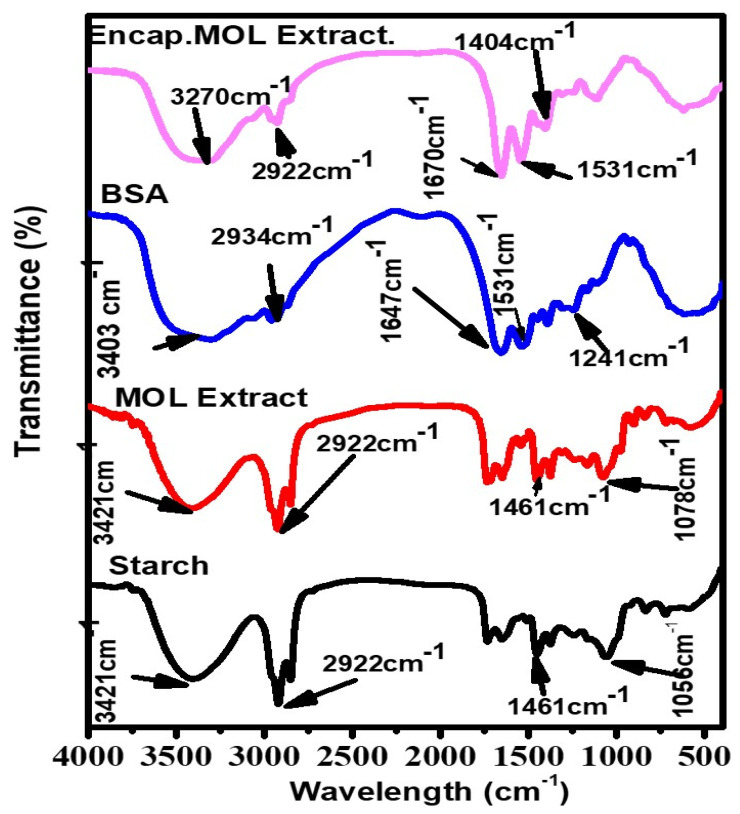
FTIR spectra of starch, MOLE, BSA and Encap. MOLE.

In Figure 4, the FTIR spectrum of starch showed a band at 3421 cm^−1^, which is associated with the stretching O-H vibration. The 2922 cm^−1^ band was related to C-H stretches due to the presence of the ring methane hydrogen atoms. Bands between 1652 to 1000 cm^−1^ were attributed to hydrogen bonds of O-H groups stretching vibration, O-H bending vibrations and C-O stretching vibrations [55].

The FTIR spectrum of MOLE showed a broad band at 2922 cm^−1^, indicating the presence of vibration stretching of the aromatic (C-H) group, while a band located at 3435 cm^−1^ belonged to (O-H) stretching vibration that was associated with phenols and alcohols. A weak band at 1461 cm^−1^ was attributed to the -OH bond. The results obtained in the present study are in agreement with [56].

In the spectrum of BSA, a broad band located at 3356 cm^−1^ and 2934 cm^−1^ can be attributed to the stretching vibration of the -NH stretch and -CH, respectively. Bands at 1647 cm^−1^ and 1531 cm^−1^ responded to C=O stretching and -N-H bending of amide I and II band [57]. Meanwhile, Encap. MOLE observed the main peaks of MOLE at 3270 cm^−1^, 2922 cm^−1^ and 1461 cm^−1^.

### 3.3. In Vivo Studies

#### 3.3.1. Liver Functions

**Figure 5:**
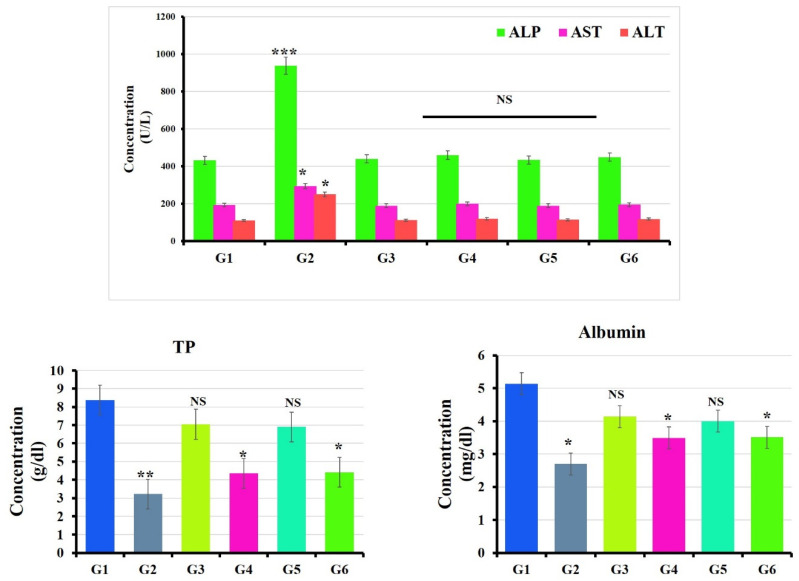
Effect of Moringa and nano-Moringa leaf extracts on liver function in Bisphenol A—Exposed versus normal rats. G1: control. G2: BPA. G3: MOLE. G4: MOLE-BPA. G5: Encap. MOLE. G6: Encap. MOLE-BPA. The data are shown as * *p* < 0.05 ** *p* < 0.01 and *** *p* < 0.001.

Animals exposed to the oral administration of BPA showed significant elevation in the levels of serum ALT, AST and ALP (*p* < 0.05) compared to the untreated group (control) [58], while a reduction in the levels of serum albumin and total protein (*p* < 0.05) were detected [59]. In contrast, hepatic function parameters were remodulated in the BPA  groups treated with MOLE and Encap. MOLE separately (Figure 5) [60].

#### 3.3.2. Oxidative Stress Markers

**Figure 6:**
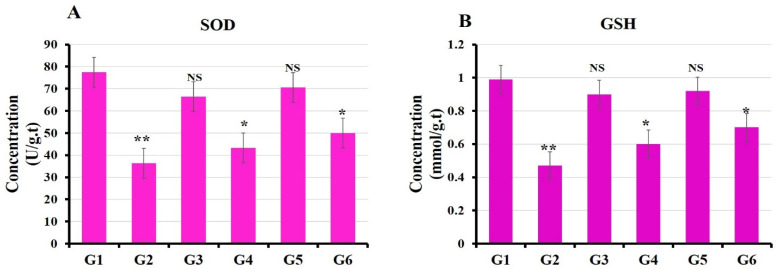
Effect of both Moringa and nano-Moringa leaf extracts on antioxidant enzyme activities in the liver tissue homogenates of Bisphenol A—Exposed versus normal rats. G1: control. G2: BPA. G3: MOLE. G4: MOLE-BPA. G5: Encap. MOLE. G6: Encap. MOLE-BPA. The data are shown as * *p* < 0.05 and ** *p* < 0.01.

In the current study, animals that were given BPA orally showed a significant decrease in the levels of GSH and SOD (*p* < 0.05) compared to the untreated group (control group) [61]. On the contrary, the levels of GSH and SOD were significantly increased in groups treated separately with MOLE and Encap. MOLE simultaneously with their exposure to BPA (Figure 6).

#### 3.3.3. Evaluations of Lipid Profile Status

**Figure 7:**
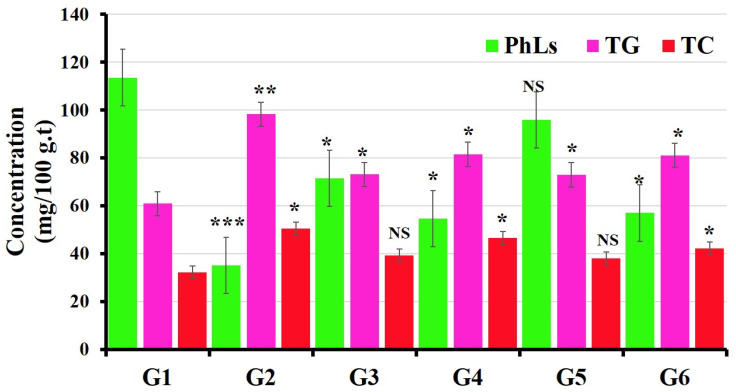
Effect of both Moringa and nano-Moringa leaf extracts on lipid profile in liver tissue homogenates of Bisphenol A—Exposed versus normal rats. G1: control. G2: BPA. G3: MOLE. G4: MOLE-BPA. G5: Encap. MOLE. G6: Encap. MOLE-BPA. The data are shown as * *p* < 0.05 ** *p* < 0.01 and *** *p* < 0.001.

In comparison to the untreated group (control group), the levels of total cholesterol (TC) and triglyceride (TG) increased significantly in the group treated with BPA [62], while an improvement in the alteration of lipid profiles was observed in the groups treated separately with MOLE and Encap. MOLE simultaneously with their exposure to BPA (Figure 7) [63].

### 3.4. ELISA Kits for Caspase-3 and Bax Detection

During apoptosis, the focal adhesion kinase, actin and poly (ADP-ribose) polymerase (PARP) were cleaved by Caspase-3 [64]. Meanwhile, endonuclease that called CAD was also activated by Caspase-3, leading to the fragmentation of DNA. Caspase-3 is regulated by bcl-2 and bcl-xL, which work to maintain mitochondrial membrane integrity and prevent the cleavage of Caspase-3 from its proenzyme state [65]. Meanwhile, the mitochondrial outer membrane permeability can be controlled by Bax, which allows the release of proapoptotic molecules (e.g., cytochrome c) [66].

**Figure 8:**
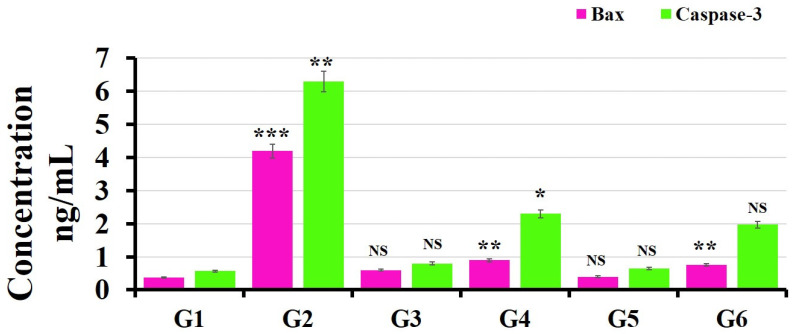
Effect of both MOLE and Encap. MOLE on the levels of Caspase-3 and Bax in liver tissues of BPA—Exposed versus normal rats. G1: control. G2: BPA. G3: MOLE. G4: MOLE-BPA. G5: Encap. MOLE. G6: Encap. MOLE-BPA. The data are shown as * *p* < 0.05 ** *p* < 0.01 and *** *p* < 0.001.

The levels of Caspase-3 and Bax in the liver tissues were significantly increased in the animal model exposed to BPA (50 mg/kg) for month by (6.3 ± 0.1 ng/mL *p* < 0.01) and (4.2 ± 0.1 ng/mL *p* < 0.001), respectively, compared to the control (0.57 ± 0.01) and (0.38 ± 0.01), respectively (Figure 8) [67]. On the other hand, groups (G4 and G6) treated separately with MOLE and Encap. MOLE during their exposure to BPA showed significant inhibition in the level of Caspase-3 and Bax by (2.3 ± 0.2 ng/mL) and (0.9 ± 0.1 ng/mL) and (1.96 ± 0.02 ng/mL) and (0.7 ± 0.04 ng/mL), respectively [68]. In contrast, the levels of Caspase-3 and Bax increased slightly in groups (G3 and G5) treated separately with MOLE and Encap. MOLE alone (0.8 ± 0.1 ng/mL) [69].

### 3.5. The Findings of Real-Time PCR

In the current study, the cyto-protective enzyme (Heme oxygenase-1 (HO-1)) was studied. This enzyme degrades heme into carbon monoxide, free iron and biliverdin, turning into bilirubin. This mechanism is important in the regulation of oxidative stress, apoptosis and inflammation. In the normal state, the expression of HO-1 is normal, while it may be increased in the pathology state due to its role in the regulation of cell homeostasis [70].

The growth arrest and DNA damage-inducible gene 45b (Gadd45b) mediates DNA damage repair, cell cycle arrest and apoptosis in response to cell injury [71]. High expression of GADD45 is used as an indicator for a variety of diseases, such as tumors [72] and nephropathy [73].

**Figure 9:**
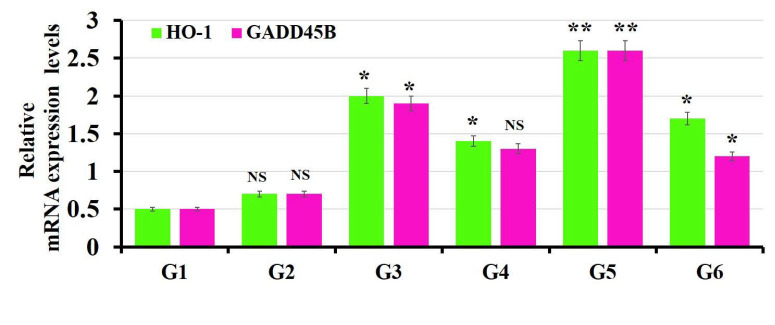
Effect of both MOLE and Encap. MOLE on the expression levels of HO-1 and Gadd45b in BPA—Exposed versus normal rats. G1: control. G2: BPA. G3: MOLE. G4: MOLE-BPA. G5: Encap. MOLE. G6: Encap. MOLE-BPA. The data are shown as * *p* < 0.05 ** *p* < 0.01 and *** *p* < 0.001.

The expression levels of HO-1 and Gadd45b increased significantly in groups (G4 and G6) treated separately with MOLE and Encap. MOLE during the course of BPA exposure compared to the untreated group (control group) (*p* < 0.05) (Figure 9) [74]. Meanwhile, MOLE and Encap. MOLE alone have the ability to maintain the homeostasis of HO-1 and Gadd45b in animal groups (G3 and G5).

### 3.6. Histopathology Results

**Figure 10:**
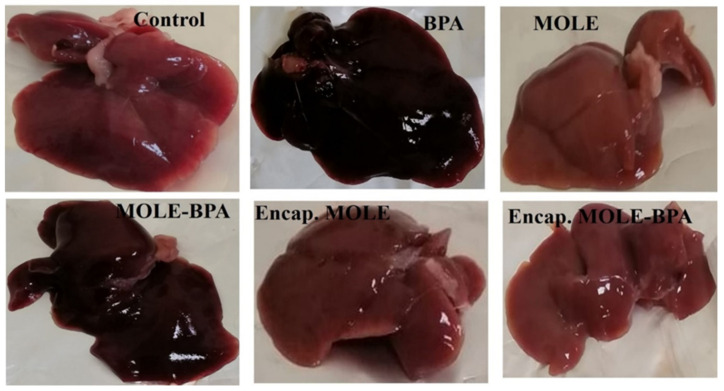
Photomicrograph of individual livers showing the morphological appearance.

**Figure 11:**
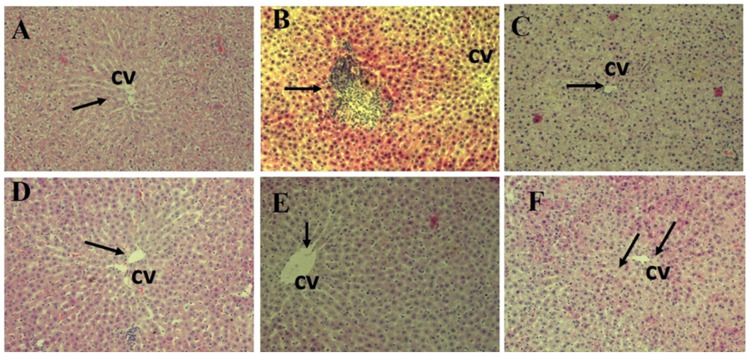
Histopathological examination of liver sections stained with H&E (200X). (**A**) Control; (**B**) BPA; (**C**) MOLE; (**D**) MOLE-BPA; (**E**) Encap. MOLE; (**F**) Encap. MOLE-BPA.

In the present study, the control group showed normal liver structure. The hepatic cords were radiating from the central vein and formed anastomosing plates that were separated by blood sinusoids and the hepatocytes are located with eosinophilic cytoplasm, central rounded and vesicular nuclei. In contrast, multiple histopathological degenerative changes in hepatic tissues were shown as a result of BPA-induced cytotoxic effect on male albino rats, including vascular dilatation and congestion, Kupffer cell proliferation, inflammatory cell infiltration and nuclear degenerative changes. These findings were consistent with the previous literature [75,76], while groups (G4 and G6) treated with MOLE and Encap. MOLE, respectively, during their exposure to BPA showed an improved histological architecture (Figure 10 and Figure 11) [77]. However, there was slight observation of vascular dilatation and blood congestion in the group (G4) treated with MOLE alone compared to G6 which was treated with Encap. MOLE. This indicates the potential therapeutic effect of *Moringa oleifera* Lam leaf extract incorporated inside hydrogel NPs. Meanwhile, an eosinophilic cytoplasm appears in both groups which could be due to the presence of isothiocyanate as a *Moringa oleifera* Lam leaf extract. For instance, isothiocyanate can produce glycosides which cause necrotic cells. This indication was clearly observed in groups (G3 and G5) that were treated separately with MOLE and Encap. MOLE, showing an eosinophilic structure and pyknotic stages [78].

**Figure 12:**
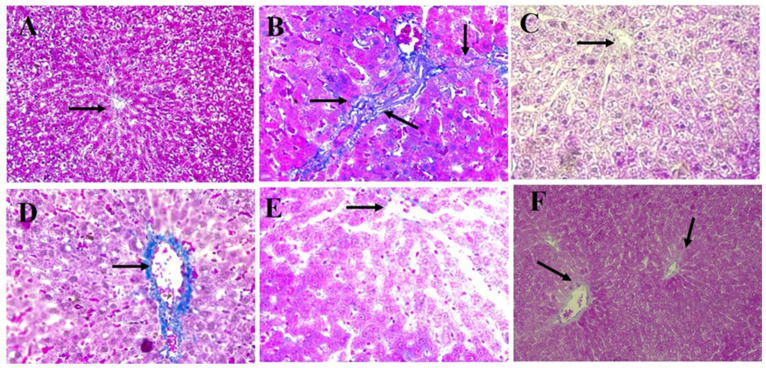
Masson Trichrome staining of liver sections (200X). (**A**) Control; (**B**) BPA; (**C**) MOLE; (**D**) MOLE-BPA; (**E**) Encap. MOLE; (**F**) Encap. MOLE-BPA.

In Figure 12, Masson trichrome stain of liver sections in the control groups revealed that collagen fibers (blue stain) were distributed normally around the central vein (CV) and portal tract (PT). However, there was an marked increase in the density of collagen fibers around the portal tract (PT) (Figure 12A–C) in the group treated with BPA [79], while encapsulation MOLE improved the distribution and density of collagen fibers during the oral administration of BPA [80]. On the other hand, collagen fibers were distributed in the portal vein area of the group treated with MOLE during the course of BPA.

**Figure 13:**
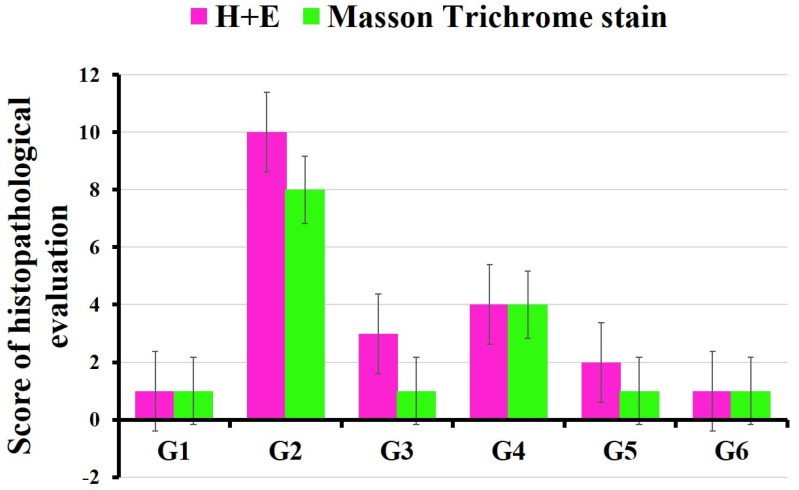
Scores of histopathological evaluation of different animal groups. G1: control. G2: BPA. G3: MOLE. G4: MOLE-BPA. G5: Encap. MOLE. G6: Encap. MOLE-BPA.

In the current study, the pathological profile of liver damage scores was calculated according to a previous publication [27]. Histopathological examination was carried out and vascular dilatation and congestion, Kupffer cell proliferation, inflammatory cell infiltration, nuclear degenerative changes and collagen fibers were evaluated. The grading scale to score pathologic findings was as follows: 0 = no injury; 1 = slight injury; 2 = moderate injury; 3 = severe injury; and 4 = very severe injury (Figure 13).

## 4. Discussion

The exposure of humans to BPA is becoming ubiquitous and continues due to its presence in components of polycarbonate plastic, dental sealant resin, flame retardants and liners for food packaging. Unfortunately, BPA is an endocrine-disrupting chemical, causing injury in the brain, liver, kidney, epididymal sperm in rodents and other organs. Xenobiotic chemical such as BPA was metabolized in the liver as the main organ for chemical detoxification. Thereby, inside the liver microsomes, BPA is glucuronidated and mediated by UGT2B1. The resultant was excreted mainly into the bile in a male rat and nonpregnant female rat. In this metabolic condition, reactive oxygen species formed [81].

For the first time, in the current study, MOLE was encapsulated inside hydrogel NPs and used to evaluate the hepatoprotective activity alongside the administration of BPA. Indeed, MOL contains antioxidants (Table 2) that can remodulate histopathological evidence induced by BPA. The physiological disorder administrated in the hepatic enzymes (ALP, ALT, AST) and in the levels of albumin and total protein was significantly remodulated in groups treated with MOLE and Encap. MOLE (Figure 5). This result indicates the hepatoprotective effect of MOLE and Encap. MOLE in eliminating free radicals that were generated by the metabolization of BPA [82,83].

The antioxidant enzymes such as SOD and GSH were significantly decreased in animals exposed to 50 mg/kg BPA, indicating that BPA strongly lowered the hepatic antioxidant status, while MOLE and Encap. MOLE improved the enzyme antioxidant activity (Figure 6). Meanwhile, the activation of Caspase-3 and Bax was significantly demonstrated in the group exposed to BPA, while MOLE and Encap. MOLE inhibited Caspase-3 and Bax levels significantly (Figure 8).

It is well known that the expression of HO-1 and GADD45B can affect oxidative stress. To provide better understanding for this hypothesis, the expression of HO-1 as a cyto-protective enzyme to maintain cell homeostasis and GADD45B as an indicator for blocking cell cycle survival, apoptosis and DNA repair were studied. In the current study, the expression of HO-1 increased significantly in groups (G4 and G6) treated separately with MOLE and Encap MOLE during the course of BPA compared to the control group (*p* < 0.05). Additionally, its expression was upregulated in groups (G3 and G5) treated with MOLE (*p* < 0.05) and Encap. MOLE (*p* < 0.001), respectively, compared to the untreated group (control group). These data were in agreement with [84], revealing the ability of MOLE to increase the expression of HO-1 (Figure 9).

Moreover, the expression of GADD45B reveals the rate of cell damage at the gene level. In the current study, a significant regulation in the level of GADD45B was obtained in groups treated with MOLE (*p* < 0.05) and Encap. MOLE (*p* < 0.001). Meanwhile, its expression was significantly reduced in the group treated with Encap. MOLE (*p* < 0.05) over the course of BPA and was reduced non-significantly in the group treated with MOLE compared to control. Nevertheless, the expression of HO-1 and GADD45B was maintained in the group that received BPA, as BPA was suspended in sesame oil, which is in agreement with [85].

The histopathology results revealed normal radial arrangement of hepatocytes along the central vein. However, hepatocytes were disordered in the group treated with BPA for a month with obvious identification of inflammatory cell infiltration. Additionally, serious eosinophilic structure was clearly shown in the cytoplasm of hepatocytes. Conversely, MOLE and Encap. MOLE significantly improved the histopathological architecture of the liver structure providing no inflammation. Moreover, collagen fibers accumulated along the portal area of the group treated with BPA, while collagen fibers were maintained in the group treated with MOLE alongside BPA and they were significantly reduced in the group treated with Encap. MOLE alongside BPA (Figure 10, Figure 11 and Figure 12).

It can be summarized that MOLE contains many antioxidant and bioactive materials that could use to protect and prevent hepatotoxicity produced by the exposure to environmentally toxic chemicals such as BPA. Encapsulation of MOLE saves its bioactive materials from temperature, humidity and enzymatic degradation. Additionally, it improves their adhesion in the small intestine, improving their adsorption.

## 5. Conclusions

M. oleifera leaf extract rich in bioactive compounds such as phenolic compounds, minerals, protein and fibers that have antioxidant capacity. However, its sensitivity to pH, temperature and other physiological enzyme degradation limits their use in biomedical applications. In the current study, phenolic compounds inserted into a starch system may alter functional properties of starch, such as gelatinization, rheological properties, gelling and retrogradation, which can improve the nutritional quality of food. Indeed, the non-covalent interactions between starch and phenolics result in either the formation of V-type amylose inclusion complex or the non-inclusive complex with much weaker binding forces. This hydrogen bridge greatly affects the hydrodynamic radius of amylose, resulting in the enhancement of phenolic compound bioavailability and control of starch digestion.

## Figures and Tables

**Table 1:** Forward and reverse primers of the selected genes.

Gene Name	Forward Primer (5′-3′)	Reverse Primer (5′-3′)
GAPDH	CTACATGGCCTCCAAGGAGTAAG	TGGAATTGTGAGGGAGATGCTC
GADD45B	GAAGATGCAGGCGGTGACTG	CCTCCTCTTCTTCGTCTATGGC
HO-1	ACAGCATGTCCCAGGATTTGTC	GGAGGCCATCACCAGCTTAAAG

**Table 2:** Identification and concentration of polyphenolic compounds in MOLE and Encap. MOLE.

Polyphenol Compounds	MOLE (µg/mL)	Encap. MOLE (µg/mL)
Gallic acid	2.5	1.44
Chlorogenic acid	9.55	5.54
Methyl gallat	0.5	0.25
Syringic acid	0.33	0.59
Pyro catechol	4.83	1.07
Rutin	3.3	2.76
Ellagic acid	26	4.09
Coumaric acid	6.87	1.08
Ferulic acid	2.97	1.49
Naringenin	4.82	3.08
Taxifolin	0.69	0.26
Cinnamic acid	0.03	0.04

## Data Availability

Data available in a publicly accessible repository.
